# 

**Published:** 1891-10-17

**Authors:** 


PRICE ONE PENNY.

No. 264, Vol. XI.—October 17th, 1891.

[Registered at the General Post Office as a Newspaper for transmission in the United Kingdom and Abroad.]

WITH EXTRA NURSING SUPPLEMENT

Per Annum, 6s. 6d.; post free

Monthly Parts, 6d.; post free 8d.

Ditto per Annum, 8s., post free.

### THE HOSPITAL

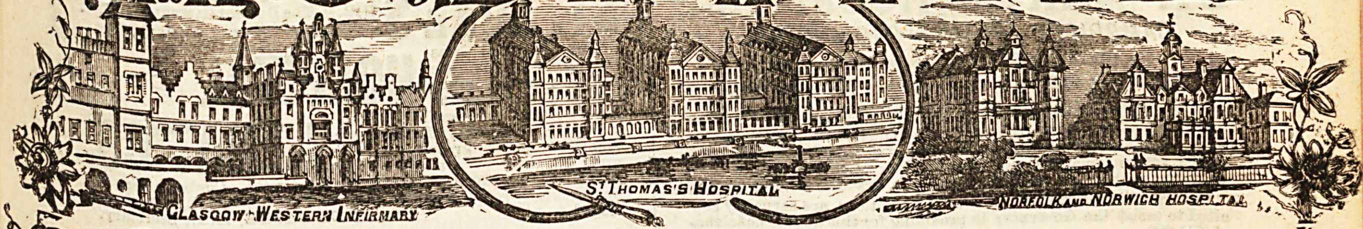

A WEEKLY JOURNAL OF

### Science, Medicine, Nursing and Philanthropy.

SATURDAY, OCTOBER 17th, 1891.

**Dr. Thorne Thorne on Diphtheria** - 25
**Passing Topics.**—The Therapeutics of Music - 26
**The Doctor's Armchair.**—Science at the Church Congress 27
**Short Trips for Short Holidays, II.**—A peep at Russia and home by Sweden - 28
**Everybody's Page** - 29
**Notes and News** - 30
**General Charities** - 31
**Annotations.**—Mr. W. H. Smith—Golden Rules—The Muzzle Wanted Again - 32
**Talks About Food.** II.—The Relation of Food to Labour (*continued*) - 36
**Scraps and Gleanings** - 31
**The Practitioner's Mirror and Retrospect—**
Medicine.—New Method of Treatment in Phthisis. III.—Cantharidinate of Potash - 33
Surgery.—Recent Investigation into the Pathology of Tetanus and the Production of Immunity in Animals - 34
Therapeutics.—Digitalis in Pneumonia - 35
Practitioners' Bookshelf.—Hydrophobia and Pasteur—The Nature and Treatment of Diphtheria—The Treatment of Typhoid Fever - 35
**The Student of Medicine.**—Appointments—Vacancies—Scholarships and Prizes—Athletics.

EXTRA SUPPLEMENT—THE NURSING MIRROR.

**En Passant.**—Uniforms—Nurses for the Middle Classes—Paying Pupils in Asylums—Short Items—Nurses for Stockport—The Row at Glasgow - xiii

**Lectures on Surgical Ward Work and Nursing.**—By Alex. Miles, M.D. Edin., F.R.C.S.E. Lecture XXXVII.—Artery Forceps - xiv

**The Nurses' Bookshelf**—The "Hospital Annual"—a Manual for Mothers—A Hint for Bazaars - xiv

**Notice** - xiv

**The Cyprus Society** - xv

**A Bed for a Sick Nurse** - xv

**For Reading to the Sick**—The Cloud - xv

**American Notes** - xvi

**Everybody's Opinion.**—Nurses' Food—Male Nurses - xvii

**Presentation—Death in Our Ranks** - xvii

**Appointment—Notes and Queries** - xvii

**Off Duty.**—From Pharmacy to Farming - xviii

**Amusements and Relaxation** - xviii

